# Supplementary material for: Mapping the evolution of fertility support policies in China: A content and instrumental analysis
Source: PLoS One. 2025 Oct 9;20(10):e0332137. doi: 10.1371/journal.pone.0332137 (PMC12510515; doi:10.1371/journal.pone.0332137)
Supplement: S1 Appendix — (ZIP) [file pone.0332137.s001.zip › S1 Appendix. 226 original policy documents/115-人力资源社会保障部、教育部等九部门关于进一步规范招聘行为促进妇女就业的通知(FBM-CLI-4-329713).doc]

人力资源社会保障部、教育部等九部门关于进一步规范招聘行为促进妇女就业的通知

**发布部门：** [人力资源和社会保障部](https://www.pkulaw.com/law/chl?Aggs.IssueDepartment=60236) [教育部](https://www.pkulaw.com/law/chl?Aggs.IssueDepartment=60203) [机构沿革](javascript:void(0))

**发布日期：**2019.02.18

**实施日期：**2019.02.18

**时效性：** [现行有效](https://www.pkulaw.com/law/chl?Aggs.TimelinessDic=01)

**效力级别：** [部门规范性文件](https://www.pkulaw.com/law/chl?Aggs.EffectivenessDic=XE0302)

**法规类别：** [招工与就业](https://www.pkulaw.com/law/chl?Aggs.Category=08702)

人力资源社会保障部、教育部等九部门关于进一步规范招聘行为促进妇女就业的通知

各省、自治区、直辖市及新疆生产建设兵团人力资源社会保障厅（局）、教育厅（教委）、司法厅（局）、卫生健康委（卫生计生委）、国资委、医保局、总工会、妇联、人民法院：
　　男女平等是我国基本国策。促进妇女平等就业，有利于推动妇女更加广泛深入参加社会和经济活动，提升社会生产力和经济活力。党和政府对此高度重视，[劳动法](https://www.pkulaw.com/chl/6393f2e43412bddbbdfb.html)、[就业促进法](https://www.pkulaw.com/chl/1e8e09abdbcff012bdfb.html)、[妇女权益保障法](https://www.pkulaw.com/chl/f20e7cd055022d39bdfb.html)等法律法规对保障妇女平等就业权利、不得实施就业性别歧视作出明确规定。当前我国妇女就业情况总体较好，劳动参与率位居世界前列，但妇女就业依然面临一些难题，尤其是招聘中就业性别歧视现象屡禁不止，对妇女就业带来不利影响。为进一步规范招聘行为，促进妇女平等就业，现就有关事项通知如下：
　　一、把握总体工作要求。各地要以习近平新时代中国特色社会主义思想为指导，深入贯彻男女平等基本国策，把解决就业性别歧视作为推动妇女实现更高质量和更充分就业的重要内容，坚持突出重点和统筹兼顾相结合，坚持柔性调解和刚性执法相结合，坚持积极促进和依法惩戒相结合，以规范招聘行为为重点，加强监管执法，健全工作机制，加大工作力度，切实保障妇女平等就业权利。

　　二、依法禁止招聘环节中的就业性别歧视。各类用人单位、人力资源服务机构在拟定招聘计划、发布招聘信息、招用人员过程中，不得限定性别（国家规定的女职工禁忌劳动范围等情况除外）或性别优先，不得以性别为由限制妇女求职就业、拒绝录用妇女，不得询问妇女婚育情况，不得将妊娠测试作为入职体检项目，不得将限制生育作为录用条件，不得差别化地提高对妇女的录用标准。国有企事业单位、公共就业人才服务机构及各部门所属人力资源服务机构要带头遵法守法，坚决禁止就业性别歧视行为。

　　三、强化人力资源市场监管。监督人力资源服务机构建立健全信息发布审查和投诉处理机制，切实履行招聘信息发布审核义务，及时纠正发布含有性别歧视内容招聘信息的行为，确保发布的信息真实、合法、有效。对用人单位、人力资源服务机构发布含有性别歧视内容招聘信息的，依法责令改正；拒不改正的，处1万元以上5万元以下的罚款；情节严重的人力资源服务机构，吊销人力资源服务许可证。将用人单位、人力资源服务机构因发布含有性别歧视内容的招聘信息接受行政处罚等情况纳入人力资源市场诚信记录，依法实施失信惩戒。

　　四、建立联合约谈机制。畅通窗口来访接待、12333、12338、12351热线等渠道，及时受理就业性别歧视相关举报投诉。根据举报投诉，对涉嫌就业性别歧视的用人单位开展联合约谈，采取谈话、对话、函询等方式，开展调查和调解，督促限期纠正就业性别歧视行为，及时化解劳动者和用人单位间矛盾纠纷。被约谈单位拒不接受约谈或约谈后拒不改正的，依法进行查处，并通过媒体向社会曝光。

　　五、健全司法救济机制。依法受理妇女就业性别歧视相关起诉，设置平等就业权纠纷案由。积极为遭受就业性别歧视的妇女提供法律咨询等法律帮助，为符合条件的妇女提供法律援助。积极为符合条件的遭受就业性别歧视的妇女提供司法救助。

　　六、支持妇女就业。加强就业服务，以女大学生为重点，为妇女提供个性化职业指导和有针对性的职业介绍，树立正确就业观和择业观。组织妇女参加适合的培训项目，鼓励用人单位针对产后返岗女职工开展岗位技能提升培训，尽快适应岗位需求。促进3岁以下婴幼儿照护服务发展，加强中小学课后服务，缓解家庭育儿负担，帮助妇女平衡工作与家庭。完善落实生育保险制度，切实发挥生育保险保障功能。加强监察执法，依法惩处侵害女职工孕期、产期、哺乳期特殊劳动保护权益行为。对妇女与用人单位间发生劳动人事争议申请仲裁的，要依法及时快速处理。

　　七、开展宣传引导。坚决贯彻男女平等基本国策，强化男女平等意识，逐步消除性别偏见。加大反就业性别歧视、保障妇女平等就业权利法律、法规、政策宣传，引导全社会尊重爱护妇女，引导用人单位知法守法依法招用妇女从事各类工作，引导妇女合法理性保障自身权益。树立一批保障妇女平等就业权利用人单位典型，对表现突出的推荐参加全国维护妇女儿童权益先进集体、全国城乡妇女岗位建功先进集体等创评活动。营造有利于妇女就业的社会环境，帮助妇女自立自强，充分发挥自身优势特长，在各行各业展示聪明才智，体现自身价值。

　　八、加强组织领导。各地区、各有关部门要高度重视促进妇女平等就业，履职尽责、协同配合，齐抓共管、综合施策。人力资源社会保障部门要会同有关部门加强对招用工行为的监察执法，引导合法合理招聘，加强面向妇女的就业服务和职业技能培训。教育部门要推进中小学课后服务。司法部门要提供司法救济和法律援助。卫生健康部门要促进婴幼儿照护服务发展。国有资产监督管理部门要加强对各级各类国有企业招聘行为的指导与监督。医疗保障部门要完善落实生育保险制度。工会组织要积极推动企业依法合规用工。妇联组织要会同有关方面组织开展相关评选表彰，加强宣传引导，加大对妇女的关心关爱。人民法院要积极发布典型案例、指导性案例，充分发挥裁判的规范、引导作用。人力资源社会保障部门、工会组织、妇联组织等部门对涉嫌就业性别歧视的用人单位开展联合约谈。

人力资源社会保障部
教育部
司法部
卫生健康委
国资委
医保局
全国总工会
全国妇联
最高人民法院
2019年2月18日

© 北大法宝：（[www.pkulaw.com](https://www.pkulaw.com/)) 专业提供法律信息、法学知识和法律软件领域各类解决方案。北大法宝为您提供丰富的参考资料，正式引用法规条文时请与标准文本核对。
欢迎查看所有[产品和服务](https://www.pkulaw.com/helps/2.html)。法宝快讯：[如何快速找到您需要的检索结果？](https://www.pkulaw.com/helps/70.html)[法宝](https://www.pkulaw.com/helps/69.html)V6有何新特色？

扫描二维码阅读原文

**原文链接：**<https://www.pkulaw.com/chl/352d0d73ec02305cbdfb.html>
